# Supplementary figures and images for: Chromoanagenesis from radiation-induced genome damage in Populus
Source: PLoS Genet. 2021 Aug 25;17(8):e1009735. doi: 10.1371/journal.pgen.1009735 (PMC8423247; doi:10.1371/journal.pgen.1009735)

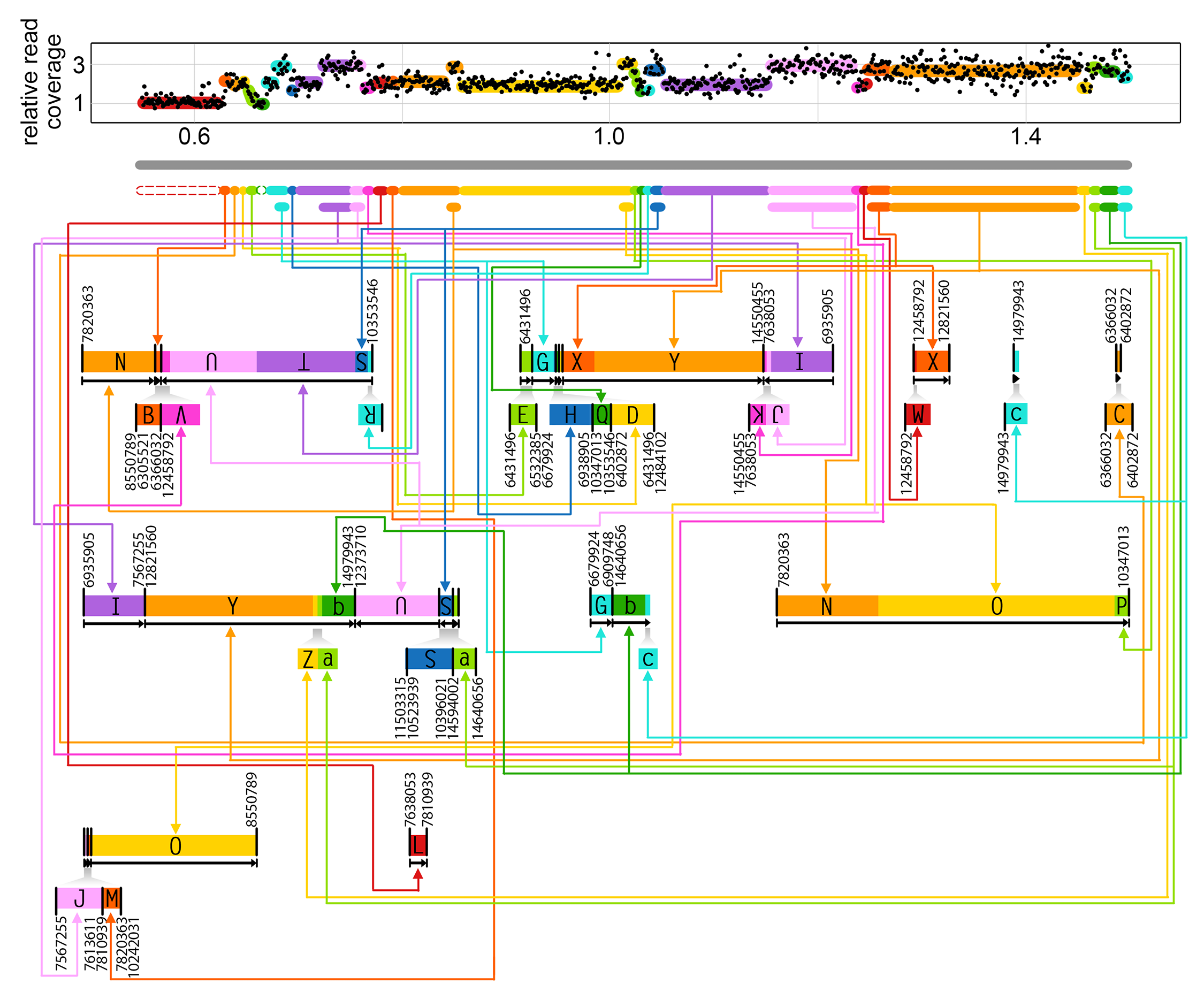

Supplement: S1 Fig — The diagram follows the same criteria as in Fig 6B. The rearranged fragments were constructed based on the data of novel junction observation in POP30_88. (TIF) [file pgen.1009735.s001.tif]

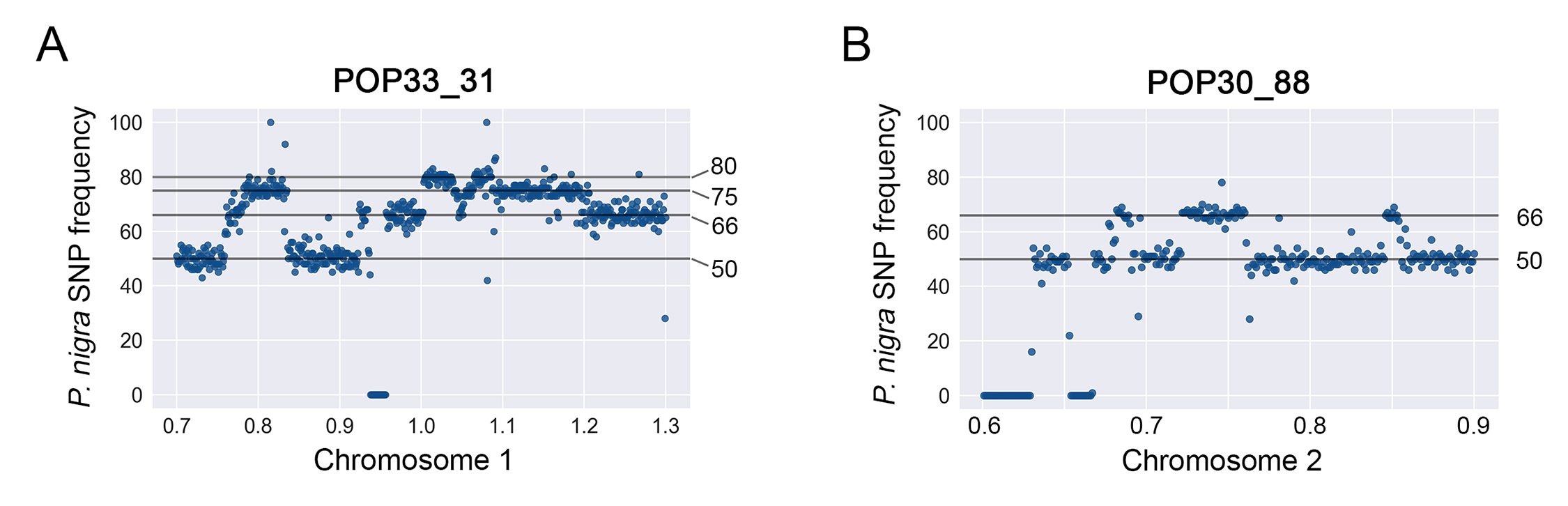

Supplement: S2 Fig — The genome was divided into consecutive non-overlapping 10kb bins. Each blue dot represents the average P. nigra SNP frequency for a 10kb bin. Horizontal lines exhibit the expected frequency levels for different copy number states, with their numbers labeled on the right. (TIF) [file pgen.1009735.s002.tif]
