# Supplementary material for: Chromoanagenesis from radiation-induced genome damage in Populus
Source: PLoS Genet. 2021 Aug 25;17(8):e1009735. doi: 10.1371/journal.pgen.1009735 (PMC8423247; doi:10.1371/journal.pgen.1009735)
Supplement: Attachment — Submitted filename: Poplar_Chromoanagenesis-Reviewer-Comments.pdf [file pgen.1009735.s009.pdf]

Reviewer 1

Considering that the repair of DNA breaks likely also occurs after zygote formation, I'm wondering whether the resulting M1 plants are chimeric. A chimeric situation could influence the outcome of the sequence analysis. To address this possibility, the authors should analyze genomic DNA isolated from individual branches of the same tree. A simple PCR with primer combinations suitable to identify rearranged DNA fragments could be used to address this concern.

Very good point, it is indeed possible that these lines are chimeric. In the course of these experiments, we have sampled all lines, including the two chromoanagenetic ones, twice. We originally sequenced the seedlings at the greenhouse. Later, after propagation into the field, we resampled one of the clones in the field. The results from genomic sequencing of these two lines were consistent with each other.

In addition, the patterns of dosage variation observed in these lines are inconsistent with chimerism. In chimeric tissue, we would expect the dosage variation to be intermediate since it would only be present in a subset of the cells. This would be diagnosed by relative coverage values intermediate between 1 and 2 for deletions, and between 2 and 3 for insertions. In the two chromoanagenetic samples, coverage values do reach 1 and 3 for deletions and insertions, respectively, consistent with homogenous samples. Similarly, the SNP frequency analysis also exhibits paternal SNP frequencies going down to 0% in its deleted regions, and reaching up to 66% in its inserted regions.

Taken together, these two elements suggest that our samples are not chimeric. We have added a supplementary figure showing the SNP frequency in the clustered CNV regions in more details (Fig. S2).

Reviewer 2

---

1. Author summary. „They". Not clear if this relates to „mechanismS" or to „type of rearrangement".

Thank you for pointing this out. We have clarified our meaning to indicate that we are talking about the rearrangements observed in these two samples. (Line 36-37)

---

2. Author summary. Helps TO understand.

Thank you. We have corrected this in the Author summary (Line 41).

---

3. Author summary. Genome instability….genome evolution (1x genome enough?) If alone standing, it is unclear what „provides a new system to characterize these types of changes." ´means. Actually, the last sentence would benefit from some more careful revision.

We have changed this sentence to the following: "<u>Characterizing such genome restructuring instances helps to understand how genome instability can remodel chromosomes and affect genome function.</u>" (Line 40-42) In the discussion, we cite Huang and Rieseberg [1], who explain the evolutionary impact of heterozygous rearrangements.

---

4. Please unify throughout: chromosome or chromosomal rearrangement.

Thank you for the reminder. We have unified the terms to "chromosomal rearrangement" (Line 211-212, changed from "chromosome rearrangement" to "chromosomal rearrangement")

---

5. Introduction. I was not really sure what to imagine under „clustered copy number variation". As this term is used further on, I suggest to define a little what is understood under „clustered" (and/or „unclustered") CNV. This can also be done by referring to an example.

This is a good point. The "clustered copy number variation" refers to more than 10 copy number changes on a single chromosome arm. We have clarified it in Results. (Line 112-113)

---

6. Introduction. „The extreme restructuring of a single chromosome (or rarely two or more)" this is later contradicted by the definition of chromoplexy (I mean single vs. two or more)

Chromoanagenesis includes three processes that are all slightly different. Chromothripsis and chromoanasynthesis almost always result in single chromosome extreme rearrangement, with a few exceptions where a few chromosomes are affected [2]. Chromoplexy always affects multiple chromosomes and the reassembled fragments are not as clustered as those resulting from the other two processes [3].

We have clarified this in the introduction as well. (Line 60-63)

---

7. Introduction. Chromoanagenesis originates from a single event (as it is written), the description of chromothripsis follows this logic by (one) „dsDNA breaks". However, one break

will not result in rearrangements of tens to hundreds segments. I consider the definition of chromoanagenesis as a single event to be most critical. While I understand the point, chromothripsis usually results from a number of events (i.e. DSBs) caused (probably) by one (major) agent/stimulus.

We have changed the sentence to the following: "<u>Chromoanagenesis results from a single triggering event that leads to highly complex segmental rearrangements</u>." (Line 50-51)

---

8. Introduction. I was unsure whether I understand what SHUFFLED chains of rearrangements mean.

We have rephrased this description as well. (Line 61-62) The "shuffled chains" represented multiple rearranged chromosomes, resulting from broken DNA ends shuffled together and religated to one another in a novel configuration [4,5].

---

9. Introduction. I do not understand to what „together covering the whole genome multiple times [19]." is related. To insertions and deletions?

The indels (insertions and deletions) that we observed in the poplar population were randomly localized across the genome. Cumulatively, every genomic position is covered by at least one indel in one of the poplar individuals.

---

10. Results. Please explain better how you came from 2 trees to 9 plants. This is somewhat confusing at present.

Based on our initial low-pass sequencing, we identified two lines with clustered CNVs on a single chromosome. In order to be able to characterize the genomes of these lines in more detail, we sequenced them deeper. We selected 7 other lines to sequence deeper as well, to serve as controls for the two chromoanagenetic lines. Of those 7 controls, three did not display any indels, and 4 displayed one or a few indels, similarly to the majority of the other trees in the population. Using these controls, we could ask whether novel junctions were associated with "regular" indels as well, or even present in genomes in which no dosage variation was visible. We did not identify novel junctions in any of the 7 controls.

To avoid confusion, we have modified our text and now only use the term "line" when referring to a specific F1 hybrid progeny (instead of "tree" or "sample" or "line").

---

11. Results. "Genomic DNAs were sent"

Thank you. We have corrected it in Results (Line 104).

---

12. Results. “individuals originated from loss or gain of the paternal P. nigra copy (Fig 2), confirming that pollen irradiation caused dosage variation” Although I understood the point, some readers may not be so clear that P. nigra donated the paternal genome in previous crosses.

We have clarified this sentence as well by indicating that the *P. nigra* pollen was irradiated. (Line 110-111)

---

13. Results. “more than 10 CNVs clustered” Then, 21 CNVs…Does this simply mean that 21 is more than 10? If yes, perhaps a range would be better…

Thank you. We have changed the sentence to the following: “<u>Both samples in the Shattering Group fit our definition of clustered changes (>10 events per chromosome arm).</u>” (Line 112-113)

---

14. Results. When starting to describe where CNVs were located, it would be good to say how many chromosomes the poplar hybrids had. This of course could be stated earlier in the text. Also, a reader is informed that the hybrids are sterile (is this really true or the trees were too young to flower?), but there is no information as why? Is this due to problems with chromosome pairing (mitosis, meiosis), whas this researched?

Thanks. We have mentioned the poplar chromosome number as follows: “Each F1 line was characterized by a unique set of indels randomly distributed along the 19 chromosomes of the poplar genome.” (Line 97-98)

As to the fertility of these lines, this is an excellent question. After several years in the field, the majority of the poplar F1 hybrids have started flowering, and both male and female flowers have been observed. We do not know whether they produce viable seed though, this is outside of the scope of our project and this publication. Poplar trees are dioecious and crosses are very difficult to perform and follow given the height of the trees when they reach sexual maturity.

---

15. Results. Unify throughout: shattering group OR Shattering Group.

Thank you for the reminder. We have unified the terms to “Shattering Group” (Line 117-118, change “shattering group” to “Shattering Group”)

16. Results. At the end of this paragraph…it is a bit difficult to follow the narrative. The difference between the two plants are insertions vs. duplications. Please make clearer why “different” the mechanisms were different.

Thanks. Of the two lines with clustered CNVs, only one exhibited deletions and duplication, resembling chromothripsis, where low copy number alteration is an essential feature [6]. In the other line, some fragments underwent triplication and quadruplication, which fits the characteristics of chromoanasynthesis [7]. By these criteria, we suggested that one sample underwent chromothripsis, and the other underwent chromoanasynthesis.

We have clarified this in the text as well. (Line 120-123)

---

17. Results. I suggest to be more specific in this section. For example, I was not able to easily see how big the insertions were compared to deletions. Another point is figure 2. If looking at fig. 2A (for instance), how can I see 2 deletions and 19 insertions? And copy number states? At least some of the events could be colored or emphasized in some other way.

Thank you for the suggestion. Yes, you are right, it is not easy to count all of the indels in Fig.2, because some of the indels are too short to be visible. We counted these indels by enlarging the plots. Our new S2 File lists all of the copy number variations and their related information.

To better show copy number variations in Fig.2, we added horizontal lines in each plot, which should clarify the indels copy number states. The number of horizontal lines were added on the right side of the plots. We have also added explanations in Fig.2 legend. (Line 547-548)

---

18. Results. Referring to fig. 4D does not comply with the definition of chromoanagenesis (two different chromosomes).

Thank you. We were accidently put the old version figure here. We have updated it and put the correct Fig.4.

---

19. Results. “Additionally, 26 (50%) breakpoints affected a gene coding sequence directly” I wonder whether the authors cannot be a little more specific what “affected” means. Considering that deletions, insertions and inversions were documented, it would be nice to spend more time on specifying the effects of these rearrangements on gene structure/function.

Here, “affected” means that the breakpoints occurred in the genic regions. To clarify the point, we have changed the sentence to the following: “<u>Additionally, 26 breakpoints (50%) occurred within a gene coding sequence and 14 of these involved gene to gene fusion</u>.” (Line 182-184) Our S4 File provides details about what genes were cut off by breakpoints and where these breakpoints are located in the genes. We believe that the data support the notion that chromoanagenesis can affect gene function. The individual outcomes are surely stochastic: on an evolutionary time scale, multiple effects are possible.

To specify the effects of rearrangements on gene structure, we added a Supplementary File 5, exhibiting the 14 breakpoints involving gene fusion.

---

20. Discussion. “For those novel DNA junctions, 1-11bp overlapping sequences were found between the joined fragments,” I understood that this was NOT always the case (see Results).

This is correct, our sentence was misleading. We just wanted to point out that junctions showing microhomology varied in the length of the microhomologous segment (1 to 11bp). The presence of microhomology at the joints is important, because it is a sign of microhomology-mediated DNA repair and non-homologous end joining [8], which supports the notion that our observed rearranged chromosomes were consistent with chromoanagenesis.

To make this part clearer, we decided to delete this sentence.

---

21. Discussion. Discussion on pages 14+15 is not very clear (but important):
I read that a sperm cell contains a micronucleus, then it is said that “during the first zygotic mitotic division, damage, such as incomplete replication, results in catastrophic DNA pulverization of the chromosome in the micronucleus”. What does this mean? Is the meaning that the micronucleus is a transient state in the sperm cell and that the same chromosome forms a micronucleus repeatedly? This is explained in the next two sentences, but not exactly.

According to the proposed mechanism of chromoanagenesis, the formation of extremely rearranged chromosomes needs at least two mitotic divisions: In the first mitosis, a broken chromosome lags during anaphase and is incorporated into a micronucleus. During the following interphase, DNA replication in the micronucleus is delayed compared with the chromosomes in the nucleus proper. In the second mitosis, the replicating micronucleus chromosome pulverizes and reassembles randomly, forming a shattered chromosome, and later reincorporates into the normal set.

Accordingly, we proposed a model to explain radiation-induced chromoanagenesis (Fig.8). Radiation was applied to binucleate pollen, which consists of a vegetative cell and a generative cell. After radiation triggers DSBs in the generative nucleus, a broken

chromosome is trapped into a micronucleus at the end of pollen mitosis 2, where the generative cell divides into two sperm cells. The micronucleus is then delivered to the egg at fertilization with the sperm. During zygotic mitosis, the micronucleus chromosome is reincorporated into the major nucleus.

We have added the summary of this information in the Discussion section. (Line 253-259)

---

22. Discussion. Also, here and earlier in the text, I was not clear on how chromoanagenesis of a single chromosome in a single (parental) genome influences pairing of homologous chromosomes? Is there data for it?

Radiation, the trigger, was applied to binucleate pollen, which had a vegetative cell and a generative cell. The chromoanagenesis process, including micronucleus formation and chromosome pulverization, did not involve meiosis, implying that no meiotic homologous chromosome pairing was involved. It is possible and even likely, that some of our poplar F1 lines will exhibit high chromosome mis-segregation at meiosis, either from the presence of the indels, or from erroneous pairing of the homoeologs in the hybrid background. As mentioned above, we do not have any data on fertility of these hybrids unfortunately. Finally, you may be referring to mitotic homologous pairing but our data does not provide any information on potential mitotic pairing either.

---

23. Discussion. Please add your thoughts on why only the two chromosomes were affected, not other. Do these two chromosomes differ from the remaining ones? Are the chromatin profiles of the two chromosomes somewhat different from the remaining ones? Such an analysis should be carried out.

We do not have sufficient instances of chromoanagenesis to tell whether there is a preference for these two chromosomes. One possible factor is selection against extensive CNVs. In both cases, the changes were compatible with satisfactory growth. Chromoanagenesis of different chromosomal regions may have deleterious effects. From the point of view of regular lesions after γ-irradiation, chromosome 1 is a good target. It displays high density of lesions, while some arms of other chromosomes have proportionally fewer lesions. But chromosome 1 is also the largest poplar chromosome, making it more likely to be affected. In the absence of further evidence, it is likely that the choice of these two chromosomes was stochastic.

---

24. Discussion. Perhaps I overlooked this in Discussion. However, I wonder why DSBs occurred in gene-rich regions in poplar and arabidopsis. Some tentative explanation in Discussion would further improve the paper. Does this mean that, for example, repetitive arrays are more resistant towards damaging gamma radiation? I am almost certain that some literature is available on the topic.

We have wondered about this very question. Besides the poplar and Arabidopsis results, we also found that high density of DSBs occurred on chromosome 17 in breast cancer [9]. Since human chromosome 17 has a high gene content, this result is consistent with our findings that DSBs occurred in gene-rich regions. Another factor is the likely difference between breaks that trigger chromanagenesis, such as arrest of the fork, and the following recombination events that lead to novel junctions. The first may not occur in genes, but the latter may be affected by open chromatin and thus prefer gene rich regions.

We have added the summary of this information in the Discussion section. (Line 270-275)

---

25. Figure 8.
Why chromothripsis, but mainly chromoanagenesis, is not thought to occur already during DNA replication before pollen mitosis II?

As we discussed in question 21, formation of a shattered pattern needs at least two mitotic divisions. Because radiation was applied to mature pollen that had already completed pollen mitosis 1, there was only one mitosis left before fertilization. So missegregation events can only occur and form micronuclei during pollen mitosis 2. We assume that the chromosome pulverization occurred in the subsequent mitosis, which was the zygotic mitosis.

Reviewer 3

Thank you for your comments. We appreciate that you enjoyed reading our report.

References

1. Huang K, Rieseberg LH. Frequency, origins, and evolutionary role of chromosomal inversions in plants. Frontiers in plant science. 2020;11:296.

2. Stephens PJ, Greenman CD, Fu B, Yang F, Bignell GR, Mudie LJ, et al. Massive genomic rearrangement acquired in a single catastrophic event during cancer development. cell. 2011;144(1):27-40.

3. Shen MM. Chromoplexy: a new category of complex rearrangements in the cancer genome. Cancer cell. 2013;23(5):567-9.

4. Baca SC, Prandi D, Lawrence MS, Mosquera JM, Romanel A, Drier Y, et al. Punctuated evolution of prostate cancer genomes. Cell. 2013;153(3):666-77.

5. Berger MF, Lawrence MS, Demichelis F, Drier Y, Cibulskis K, Sivachenko AY, et al. The genomic complexity of primary human prostate cancer. Nature. 2011;470(7333):214-20.

6. Korbel JO, Campbell PJ. Criteria for inference of chromothripsis in cancer genomes. Cell. 2013;152(6):1226-36.

7. Pellestor F, Gatinois V. Chromoanasynthesis: another way for the formation of complex chromosomal abnormalities in human reproduction. Human reproduction. 2018;33(8):1381-7.

8. Hastings PJ, Ira G, Lupski JR. A microhomology-mediated break-induced replication model for the origin of human copy number variation. PLoS genetics. 2009;5(1):e1000327.

9. Przybytkowski E, Lenkiewicz E, Barrett MT, Klein K, Nabavi S, Greenwood CM, et al. Chromosome-breakage genomic instability and chromothripsis in breast cancer. BMC genomics. 2014;15(1):1-5.
